# The relationship between organizational commitment and turnover intention among temporary employees in the local government: Mediating role of perceived insider status and moderating role of gender

**DOI:** 10.3389/fpsyg.2022.1024139

**Published:** 2022-12-16

**Authors:** Defeng Xia, Jingru Sun, Congcong Zhang, Yiying Zhang

**Affiliations:** ^1^School of Politics and Public Administration, Zhengzhou University, Zhengzhou, Henan Province, China; ^2^Research Center for Contemporary Capitalism of Zhengzhou University, Zhengzhou, Henan Province, China; ^3^School of Education, Zhengzhou University, Zhengzhou, Henan Province, China

**Keywords:** turnover intention, temporary employees, perceived insider status, local government, organizational commitment

## Abstract

**Purpose:**

The purpose of this study is to examine the relationships between organizational commitment and turnover intention, and to test the mediating effect of perceived insider status, and moderating effect of gender on that relationship.

**Methodology:**

Data were collected using a questionnaire survey method from 820 temporary employees of government agencies working in China. The data obtained were analyzed according to the moderated mediation.

**Findings:**

As a result of the analysis, it was determined that perceived insider status has a partial mediation effect on the relationship between organizational commitment and turnover intention. Also, the results supported the moderated mediation and showed that the indirect effect of organizational commitment and turnover intention through perceived insider status was weaker for males than females. Then, the theoretical and practical implications of the findings are discussed.

## Introduction

With the continuous development of the economy and society, the government has taken on more and more responsibilities in economic construction, social management, people’s livelihood, and public services, which has placed higher demands on the ability and efficiency of government departments to perform their duties (Joo et al., 2015). Under the general policy of “no increase but a decrease” in the number of financially-supported staff, the existing stable staffing management can hardly meet the needs of the increased responsibilities. Nonrenewable fixed-term and temporary agency employment contracts began to gradually replace the traditional model of long-term employment, and the proportion of temporary employees in China has continuously increased in recent years (Liu, 2015; Duan et al., 2021; Liu X. et al., 2022). The temporary supernumerary staff has become an important way and channel to make up for the shortage of human resources in government agencies and institutions at all levels (Conway et al., 2016).

Temporary employees in the local government refer to workers temporarily hired by the government through public finance to assist government departments and agencies in public management activities or administrative affairs, and these workers are not government workers with a formal establishment (Lapalme et al., 2009). We need to acknowledge the complexity underlying the concept of temporary workers in China (Duah et al., 2015). Due to the limitations of government financial resources and the number of formal staff, there are a large number of temporary personnel in China’s government agencies to cope with the huge and complex social management needs. The existence of temporary workers can be considered a cost-cutting, human resource-management tool for solving unemployment. Temporary government employees in the government can well alleviate the plight of insufficient personnel in formal bureaucratic organizations, and make important contributions to the normal operation of government departments and social development. However, temporary workers also receive less protection and more job insecurity (Balz, 2017; Guo et al., 2022). Considering the low-level work and low wage guarantees, there are problems of weak organizational commitment and high turnover, and the difference in employment status affects the stability of employment relationships. Thus, it is of great significance to investigate the turnover intention and influencing factors of temporary employees in the government to maintain the operation of formal bureaucracy and social management.

We review the existing studies on temporary organizations or temporary employees. Some studies try to explain the growth of temporary work in China from the perspective of institutional and organizational characteristics (Liu, 2015). Some studies focus on the differences in work behavior between temporary employees and permanent employees, like engaging in deviant behaviors, employee performance, and turnover intentions (Nuhn et al., 2018; Liu X. et al., 2022). In addition, there is a lot of research using the framework of social exchange theory, self-determination theory, psychological compensation, and social identity theory to investigate the impact of organizational identity, and job insecurity on work attitudes, work engagement, and so on (Slattery et al., 2010; Jiang and Wang, 2018; Zhang et al., 2018; Duan et al., 2021; Guo et al., 2022; Jing and Yan, 2022; Liu X. et al., 2022). However, the use of temporary employment has increased in local government, but few studies to date have analyzed the turnover intentions of temporary employees in the local government (Liu X. et al., 2022). Studies conducted on the turnover intention of government temporary have mainly sorted out the management of government temporary personnel from the macro aspect (Hu, 2015).

The purpose of this paper is to combine social identity, social exchange, and role conflict theories into a model explaining the turnover intentions of temporary employees in the local government from an individual level. Therefore, this study tries to investigate the relationship between organizational commitment and turnover intention, as well as the mediating role of perceived insider status and the moderating role of gender, to reduce the detrimental effects of turnover intentions from temporary employees, promote their motivation, and provide informative policy recommendations for improving the management of government temporary staff.

## Literature and hypotheses

### Organizational commitment and turnover intention

Organizational commitment is linked with a strong desire amongst employees to remain within their organizations, the identification and reliance of employees on organizational goals, values, etc., as well as their internal driving force to stay in the organization and an important manifestation of their loyalty to the organization (Porter et al., 1973; Loan, 2020; Nisar and Rasheed, 2020; Novianti, 2021). Three dimensionalities of organizational commitment include affective commitment, continuous commitment, and normative commitment (Allen and Meyer, 1990; Meyer and Allen, 1991). Affective commitment is the most fundamental of these elements, which is related to the individual’s recognition and evaluation of his status and value, and whether he has a sense of identity and commitment to his organization. Organizational commitment is an internal subjective consciousness, which is derived from the effect of the organizational environment on people, and the result of this effect will further affect the behavior of people in the organization (Belle et al., 2015).

Following social identity theory, temporary employees usually perceive high job insecurity, which concerns the perception of an involuntary and undesired change in the continuity of the work situation (Balz, 2017; Liu X. et al., 2022). Previous studies have shown that organizational commitment is an important factor affecting turnover intention. Having a sense of organizational commitment will promote employees to work hard in the organization and reduce turnover intention (Chung et al., 2017), the lack of commitment will reduce employees’ efforts to work and lead to the emergence of the propensity to leave (Boros and Curşeu, 2013; Joo et al., 2015; Shaikh et al., 2022). Research on Chinese enterprises and state-own enterprises supports the correlation between organizational commitment and employees’ willingness to leave (Jing and Yan, 2022; Liu X. et al., 2022). In light of the evidence reviewed in this section, we propose the following hypothesis:

*Hypothesis 1*: The organizational commitment of government temporary employees negatively correlates with their turnover intention.

### Organizational commitment and perceived insider status

Perceived Insider Status was first proposed by Stamper and Masterson, is defined as the extent to which an individual employee perceives him or herself as an insider within a particular organization, describing employees’ cognition of their identity status in the organization, feeling accepted and recognized by the organization, and truly becoming the “internal people “of the organization (Stamper and Masterson, 2002; Dai and Chen, 2015; Koçak, 2020; Xiao et al., 2020; Liu D. et al., 2022). An important view in insider identity cognition research is to explain the employee-organization relationship based on the social exchange theory, arguing that differences in organizational attitudes perceived by employees will have an important impact on employee performance or other outputs (Liu D. et al., 2022). There is a huge difference in the identities of casual and regular workers (Knapp et al., 2014). Researches show that perceived insider status has a strong effect on employees’ job performance and organizational citizenship behavior. When employees continue to gain support and trust from the work, they actively see themselves as the owners of the organization, which is the process of insider status perception. On the other hand, employees with a strong sense of organizational commitment will have a stronger identification with organizational values and organizational behavior. When employees consider that the organization cherishes them, they will have a deeper perception of acceptance they have obtained in the organization (Wang et al., 2021; Guo et al., 2022). Therefore, employees’ sense of organizational commitment will significantly affect their perceived insider status. Based on the evidence relating to organizational commitment and perceived insider status, we propose the following hypothesis:

*Hypothesis 2*: The organizational commitment of government temporary employees positively correlates with their perceived insider status.

### Perceived insider status and turnover intention

Perceived insider status can be affected by personal and organizational factors, which in turn can affect employee behavior in the organization. Relevant studies have investigated the role of perceived insider status as a predictor of attitudinal and behavioral work outcomes, especially in the relationship between individuals and organizations (Raub, 2016; Chen et al., 2017). Once new employees perceive themselves to be seen as organizational insiders, they have a strong sense of belonging, actively work for the organization, and do not want to leave the organization. In a highly collective cultural society, obtaining personal space and insider status is the precondition that Chinese employees devote themselves to work. Previous research results have confirmed that employees’ perceived insider status is negatively correlated with their turnover intention (Knapp et al., 2014). Employees’ willingness to stay in the organization is largely influenced by their level of involvement in the organization and the extent to which the organization recognizes them (Shao et al., 2022). Compared with permanent employees, temporary workers have a lower sense of insider status and are more likely to leave their jobs because they worry about their future jobs and their low status among members (Arain et al., 2018; Liu X. et al., 2022). Based on this information, the following hypothesis has been developed:

*Hypothesis 3*: Perceived insider status may affect turnover intention negatively.

### The mediating role of perceived insider status

Combining the three hypotheses above, it is easy to find that the impact of organizational commitment on the turnover intention of government temporary employees may partially affect the identity of insiders. Studies have shown that after the needs of employees in the organization are met, they will have a sense of attachment to the organization, so their willingness to continue to maintain membership in the organization will be stronger, and the generation of turnover behavior will be reduced (Knapp et al., 2014). The higher the employee’s sense of identity in the organization, the stronger the insider’s identity, the more willing they are to stay in the organization, and the lower the turnover intention (Oh, 2019). Based on the above predictions of this paper, the relationship between organizational attachment and their propensity to leave among government temporary employees may be significantly mediated by a certain factor, which may be the perception of insider status (Slattery and Rajan Selvarajan, 2005; Caron et al., 2019). This conjecture also reveals the deeper mechanism of how organizational commitment affects turnover intention. Considering the explanations, Hypothesis 4 is formed as follows:

*Hypothesis 4*: Perceived insider status may mediate the relationship between organizational commitment and turnover intention.

### The moderating role of gender

Turnover intention is a complicated manifestation, which is a comprehensive performance of job dissatisfaction, leaving thoughts, looking for other jobs, and the possibility of finding other jobs (Boros and Curşeu, 2013; Flickinger et al., 2016; Jing and Yan, 2022). In addition to the factor of perceived insider status, the influence of individual factors on turnover intention should also be considered. Several studies have suggested that the relationship between gender should affect the relationship between our variables of interest because men generally attain a higher status and potentially more fulfilling roles in organizations (Russ and McNeilly, 1995). Due to the different social expectations of male and female roles, traditional women are more played family roles and more men play characters still affects the positioning of characters (Kerr and MacCoun, 1985). Society’s expectations for different genders make each gender align with their behavior patterns (Bussey and Bandura, 1999). Therefore, men’s and women’s sense of belonging to the organization, resignation behavior, and resignation tendencies are also different. If gender acts as a moderator, a major reason may be the recurring finding that women place greater importance on social relationships.

Combining gender role stereotypes and role conflict theory, men feel a strong sense of insider identity and have a lower turnover intention when they have a high sense of organizational commitment, which is inseparable from gender roles. Society’s perception of women is that family attributes are stronger than work attributes. In addition, women take on more responsibility for caring for the family and raising children than men (Petrongolo, 2019). Studies have found that compared with men, women are more vulnerable to family burdens, which limits their commitment to work (Camgoz et al., 2016; Lee et al., 2017). Therefore, under the same high level of organizational commitment, women may still have a higher turnover intention due to the influence of perceived insider status. Thus, we propose the following hypothesis:

*Hypothesis 5*: Gender plays a moderating role between perceived insider status and turnover intention.

**Figure 1:**
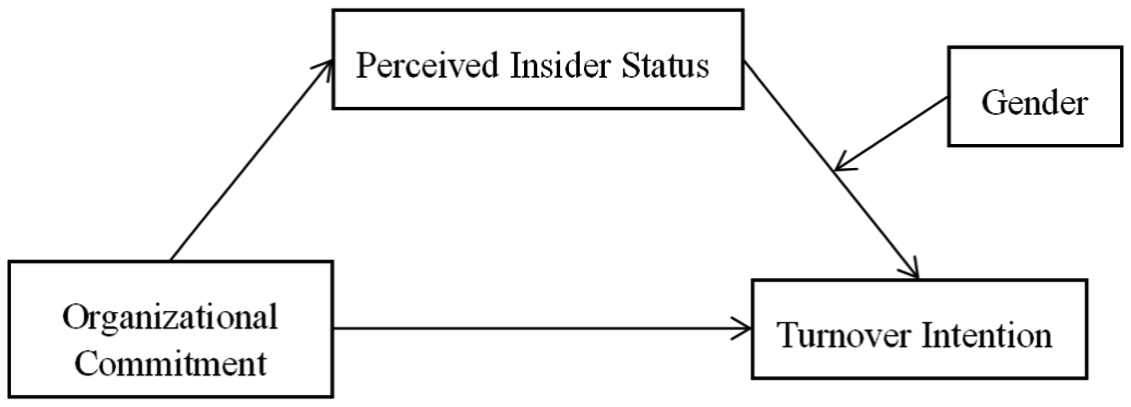
Theoretical framework.

The theoretical framework of this study can be summarized as follows (see Figure 1).

## Materials and methods

### Sample and procedure

In this study, questionnaires were distributed to temporary employees of government agencies in Henan Province through Questionnaire Star (restricting the terminals fulfilling in the questionnaires through IP addresses), and a total of 840 questionnaires were recovered with 820 valid questionnaires obtained, and the effective recovery rate was about 97.62%. Among them, as shown in Table 1: (1) The number of male samples is more than that of women, accounting for about 55.61%; (2) the number of samples in the 21–30-year-old stage is the largest, accounting for about 54.15% of the total number of samples, followed by 31–40 years old, accounting for 30.24%, which is also in line with the actual situation of this group; (3) the number of samples with undergraduate and college degrees There are many people, accounting for 45.85 and 33.17% of the total sample respectively, and only 16.59% of the graduate students and above. It can be seen that the educational background of this group of people is mostly at the undergraduate level or below; (4) in terms of working time in the current agency, more than 70% belong to <3 years, of which 1–3 years account for about 50.24% of the total sample size, <1 year accounted for 20.98%, 3–5 years accounted for 18.54%, and more than 5 years accounted for only 10.24%. Most government temporary employees have a relatively short working life in the current unit.

**Table 1:** Sample description.

Demographic variable	*N* (%)
*Gender*	
Male	456 (55.61%)
Female	364 (44.39%)
*Age*	
≤20	16 (1.95%)
21–30	444 (54.15%)
31–40	248 (30.24%)
≥40	112 (13.66%)
*Education level*	
Senior high school or less	36 (4.39%)
College	272 (33.17%)
Undergraduate	376 (45.85%)
Postgraduate and above	136 (16.59%)
*Years of work*	
≤1 year	172 (20.98%)
1–3 years	412 (50.24%)
3–5 years	152 (18.54%)
≥5 years	84 (10.24%)

### Measures

In this study, measuring scale items were adopted from the existing literature and adapted into the Chinese context with the help of bilingual researchers and m-government experts. Before conducting the survey, we pilot-tested 50 citizens to make sure of the logical consistency, wording, meaning, and appropriateness of the instruments. All items were measured using a 5-point Likert scale ranging from “strongly disagree = 1” to “strongly agree = 5.”

Organizational Commitment. Three-Factor Organizational Commitment Scale compiled by Allen and Meyer (1990) was slightly modified according to the characteristics of the research subjects. The scale consists of three parts. The first part is the question of emotional commitment, with 4 items including “I have deep feelings for the unit” and “I would like to make any contribution to the unit”; the second part is the question of continuous commitment, there are 4 items such as “I continue to work in this unit because the benefits here are good,” “I feel that there are great opportunities for promotion here, and it is beneficial to me to stay here”; the third part is Normative commitment, including “I have responsibilities and obligations to the unit,” “I think job-hopping is immoral” and other 4 items, a total of 12 questions. The overall Cronbach’s alpha coefficient of organizational commitment was 0.813, and the Cronbach’s alpha coefficients of emotional commitment, continuous commitment, and normative commitment were 0.758, 0.685, and 0.615, respectively, all between 0.6 and 0.8.

Perceived Insider Status. The 6-item scale developed by Stamper and Masterson (2002) was translated and adjusted by Chen and Aryee (2007) in a Chinese context-oriented study, which showed that the scale has high reliability and is more practical in the Chinese context. The scale includes 3 positive scoring items, “I feel strongly that I am a member of this work unit,” and three negative scoring items, “I feel that I am an outsider in this work unit.” Cronbach’s alpha coefficient for perceived insider status is 0.871.

Turnover Intention. The 4-item scale compiled by Farh et al. (2007) was used, and some modifications were needed in combination with this study. There are 3 positive scoring questions including “I often think about quitting my current job,” and 1 reverse scoring question “I plan to do long-term career development in this unit.” Cronbach’s alpha coefficient for turnover intention is 0.889.

In this study, SPSS statistical software was used to analyze the data. A descriptive analysis was performed to examine the characteristics of the samples. A correlation analysis was performed to examine the correlations between each variable, and an effective method was used to examine causal relationships and moderating effects in studies focused on our topic.

## Results

### Reliability and validity

The Cronbach’s alpha of each variable was measured to test the reliability of the variables, all of the Cronbach’s alpha values are above 0.70, indicating high instrument reliability. The KMO values of the organizational commitment scale perceived insider status scale, and turnover intention scale is all >0.7, *p* < 0.05, the results indicate that there is a correlation between variables, suitable for factor analysis, and the questionnaire has better structural validity. Collecting data by the self-report method may lead to common method bias. Therefore, this study first used Harman’s single-factor test to verify common method variance. It was found that there were 4 factors with eigenvalues >1 in the unrotated case, and the largest factor varies nicely explained the degree of accuracy was 38.79% (<40%), so there is no serious common method variance in this study.

In addition, to ensure the validity of the regression analysis results, Collinear Diagnostics was used to test the multicollinearity problem between independent variables, and measure the tolerance and variance inflation factor (VIF) indicators. Tolerance and variance inflation factor are reciprocal to each other, the smaller the VIF, the weaker the multicollinearity, if the VIF is ≥10, it indicates that there is a very serious multicollinearity between the independent variables, it is generally believed that VIF should not be >5. In the regression analysis of this study, the VIF of the independent variables was <5, indicating that there was no multicollinearity between the variables and that the questionnaire validity was good.

### Hypotheses testing

Table 2 provides the descriptive statistics and correlation among key variables. As seen in Table 2, each variable was significantly correlated with the others. It is seen that organizational commitment is negatively correlated with turnover intention, and positively correlated with perceived insider status In addition, perceived insider status is negatively correlated with turnover intention. This result provides preliminary evidence for our model.

**Table 2:** Summary statistics and correlation matrix.

	*M*	*SD*	1	2	3	4	5	6
1. Organizational commitment	2.596	0.401	1					
2. Emotional commitment	2.831	0.591	0.834**	1				
3. Continued commitment	2.465	0.519	0.776**	0.406**	1			
4. Normative commitment	2.493	0.401	0.764**	0.498**	0.431**	1		
5. Perceived insider status	2.703	0.506	0.812**	0.677**	0.672**	0.566**	1	
6. Turnover intention	3.449	0.648	−0.812**	−0.652**	−0.725**	−0.536**	−0.799**	1

*p < 0.05, **p < 0.01, ***p < 0.001.

### Test of mediation

According to Hayes (2013) and Wen and Ye (2014), Model 4 of the SPSS macro program PROCESS is used to test the mediating effect of perceived insider status in the relationship between organizational commitment and turnover intention.

Results in Table 3, regression analysis showed that organizational commitment not only directly predicted turnover intention (*H*1; *B* = −0.80, *p* < 0.01) but also indirectly through perceived insider status; organizational commitment had a positive effect on perceived insider status (*H*2; *B* = 0.84, *p* < 0.01); perceived insider status significantly and negatively predicted turnover intention (*H*3; *B* = −0.46, *p* < 0.01). Based on the Bootstrap method using 5,000 bootstrap sample indicate that perceived insider status mediated significantly and partially between organizational commitment and turnover intention (*H*4; *B* = −0.38, *p* < 0.01, at 95%LLCI = −1.09, ULCI = −0.57).

**Table 3:** Regression analysis results of the mediating effect.

			*B*	*SE*	*t*	*p*
The direct effect of OC on TI			−0.80	0.10	−8.08	0.000
OC has a positive effect on PIS			0.84	0.06	13.63	0.000
PIS has a negative effect on TI			−0.46	0.08	−5.49	0.000
	**Value**	***SE***	**LL95%CI**	**UL95%CI**		
Indirect effect	−0.38	0.09	−0.55	−0.21		
	**M**	***SE***	**LL99%CI**	**UL99%CI**		
Bootstrapped indirect effect	−0.81	0.13	−1.09	−0.57		

*N* = 820; Bootstrap sample size = 5,000; CI, confidence interval; LL, lower limit; UL, upper limit; OC, organizational commitment; PIS, perceived insider status; TI, turnover intention.

### Test of moderated mediation

**Figure 2:**
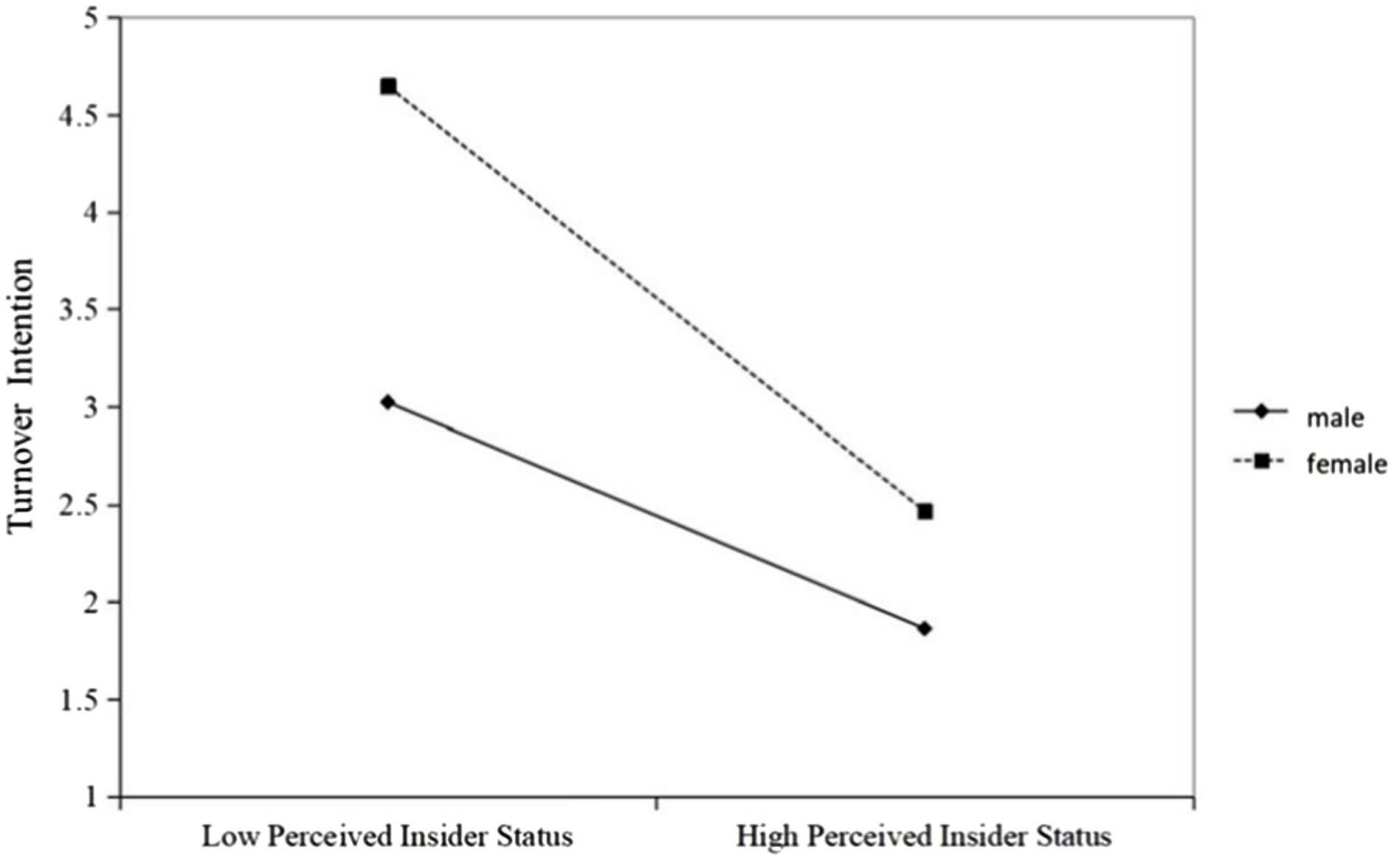
The moderating effect of gender.

Hypothesis 6 predicted that gender will play a moderating role between perceived insider status and turnover intention. The moderating effect is tested using the Model 14 of the SPSS macro program PROCESS. The results in Table 4 show that the effect of perceived insider status and gender interaction (PIS × G) on turnover intention was significant (*B* = −0.26, *p* < 0.05). To further explain the moderating effect of gender, a simple slope test was performed for both male and female groups. Figure 2 was formed using the data obtained to determine whether the effect of perceived insider status on turnover intention shows significant differences according to the different gender. As a result of the investigations, it was found that the relationship between perceived insider status and turnover intention was weaker for males than for females. The conditional indirect effect refers to the significant change of indirect effect according to the different levels of gender. Besides when the results of the model index are examined, the moderated mediation index is significant due to not containing 0. To say that Hypothesis 5 is fully supported, when the gender was male, the mediating effect value was −0.28; while when the gender was female, the mediating effect value decreased to −0.50.

**Table 4:** Regression results for moderated effect.

Predictor	*B*	*SE*	*t*	*p*
*DV = perceived insider status*				
Organizational Commitment	0.86	0.06	13.97	0.0000
*DV = turnover intention*				
Perceived insider status (PIS)	−0.06	0.18	−0.35	0.729
Gender (G)	0.56	0.29	1.91	0.058
PIS × G	−0.26	0.11	−2.37	0.019
**Gender**	**Boot indirect effect**	**Boot *SE***	**Boot *z***	**Boot** ***p***
*The conditional indirect effect on gender*				
Male	−0.28	0.09	−3.19	0.000
Mediated intermediary	−0.23	0.11	−8.39	0.000
Female	−0.50	0.11	−5.98	0.000

DV, dependent variable.

## Discussion and implications

### Discussion

Firstly, the results show that organizational commitment of government temporary employees is significantly negatively correlated with turnover intention. Sustained commitment refers to an awareness of sunk costs and a willingness to stay in an organization as individuals increase their commitment to the organization. The data of this study shows that more than 50% of the government temporary employees have worked in the unit for 1–3 years. Most of the temporary employees’ wages are only higher than the local minimum wage and are half of the formal civil servants (Yang and Yang, 2022). Due to the short working time and low wages, they are aware of relatively few sunk costs, which leads to a low sense of continuous commitment for this group of people. Emotional commitment is the recognition and acceptance of organizational goals and values by employees. Most of the team-building activities and training activities in the unit do not include temporary employees, which to a certain extent leads to the low emotional commitment of this group of people to the organization (Avanzi et al., 2014). Normative commitment means that employees feel an obligation and responsibility to remain in the organization. However, 54% of government temporary employees are between the ages of 21 and 30, most of them are young employees, and they are less constrained by traditional thinking (Liu X. et al., 2022), so they are less affected by normative attribution when choosing to leave. Organizational commitment increases with promotion, but government temporary employees are mostly in lower-level jobs that are temporary and have poor career prospects (Wu, 2021). It is very difficult for non-staff temporary employees to get a promotion.

Secondly, the perceived insider status of government temporary employees has a partial mediating effect. That is to say, the organizational commitment of government temporary employees can not only directly affect the turnover intention, but also affect the turnover intention through the intermediary role of perceived insider status. Government temporary employees’ perception of their insider identity will directly affect their turnover intention. When government temporary employees have a strong sense of belonging to the organization, they will take the initiative to regard themselves as the owners of the unit, and are more willing to stay in the organization (Jiang and Wang, 2018).

Thirdly, gender plays a moderating role between perceived insider status and turnover intention. When the staff is male, the perceived insider status has a significant negative predictive effect on their turnover intention; while when the temporary recruit is female, the perceived insider status hurts their turnover intention. Enhance the predictive effect. Therefore, it shows that under the same level of perceived insider status, different genders have different negative effects on turnover intention, that is, under the same insider identification result, women are more likely to have turnover intention. The reason for this result may be that the social cognition and role positioning of different genders are different, and men and women act according to the behavioral standards given by society, thus forming different behavioral patterns of the sexes, which are also manifested in psychological cognition. Influenced by traditional Chinese gender concepts, women tend to identify more with family roles, while men are more identified with work roles (Lee et al., 2017; Liu and Ngo, 2017).

### Conclusion and research implications

In general, this research tried to enrich the understanding of underlying mechanisms between organizational commitment and turnover intention among temporary employees in local governments in China. This paper investigated the relationships between organizational commitment and turnover intention and tested the mediating effect of perceived insider status, and moderating effect of gender on that relationship using a questionnaire survey method from 820 temporary employees of government agencies working in China. As a result of the analysis, it was determined that perceived insider status has a partial mediation effect on the relationship between organizational commitment and turnover intention. Also, the results supported the moderated mediation and showed that the indirect effect of organizational commitment and turnover intention through perceived insider status was weaker for males than females. Moreover, some suggestions for governments to decrease the turnover of their employees may be as follows.

For managers and organizations, this study suggests some practical implications. First, research findings revealed that turnover intention and organizational commitment are directly affected by organizational commitment. Managers can increase emotional commitment by taking into account temporary employees’ emotional needs, creating a positive, relaxed, and pleasant organizational atmosphere and environment. At the same time, a career management system in line with the actual situation of government temporary employees might be considered, to help temporary employees break the promotion “ceiling” and enhance their continued commitment. In addition, a variety of training activities are carried out to improve work skills, relieve work pressure, adjust work mentality, and improve the sense of normative commitment of temporary employees.

Second, research findings demonstrate that perceived insider status negatively predicts the turnover intention of government temporary employees and mediates the relationship between organizational commitment and turnover intention of government temporary employees. Therefore, government managers can positively contribute to the decrease of turnover intention, increasing perceived insider status by enhancing the sense of team support, giving respect and trust, and helping government temporary employees truly participate in organizational decision-making.

In addition, this study also highlighted the indirect effect of organizational commitment on turnover intention through perceived insider status is strong when the gender is female. Therefore, managers may pay more attention to the characteristics and needs of different genders, like flexible working hours, and family support plans. Also, some support may be helpful to get closer to employees to understand the psychological demands and expectations of employees of different genders and integrate the pursuit and development of temporary employees.

### Limitations and future research

In summary, the results of this study verify the hypothesis and have a certain value for systematically understanding the relationship between organizational commitment and turnover intention of government temporary employees, but there are still some deficiencies, and follow-up research can be improved in the following aspects: The first limitation of this study is the selection of research samples, which are affected by objective factors such as time and geographical factors. Therefore, future research should fully consider regional differences to improve the representativeness of the sample. Furthermore, the turnover intention is expressed as the psychological tendency of employees to leave their current work, which reflects the two-way interaction between temporary employees and the organization. However, because the behavior process of the organization is more complicated to measure, the research on turnover intention is only from the perspective of temporary employees. In the future, the research should be further enriched and developed from the perspective of both temporary staff and the government.

## Data availability statement

The raw data supporting the conclusions of this article will be made available by the authors, without undue reservation.

## Author contributions

DX conceived of the presented idea. JS developed the theory and performed the computations. YZ verified the analytical methods. CZ supervised the findings of this work. All authors contributed to the article and approved the submitted version.

## Funding

This work was supported by the China Postdoctoral Science Foundation (no. 2022M712858), National Social Science Fund of China (no. 22BZZ066), and Education Department of Henan Province (nos. 2023-YYZD-24 and 2021-CXTD-07).

## Conflict of interest

The authors declare that the research was conducted in the absence of any commercial or financial relationships that could be construed as a potential conflict of interest.

## Publisher’s note

All claims expressed in this article are solely those of the authors and do not necessarily represent those of their affiliated organizations, or those of the publisher, the editors and the reviewers. Any product that may be evaluated in this article, or claim that may be made by its manufacturer, is not guaranteed or endorsed by the publisher.
